# Natural computation meta-heuristics for the in silico optimization of microbial strains

**DOI:** 10.1186/1471-2105-9-499

**Published:** 2008-11-27

**Authors:** Miguel Rocha, Paulo Maia, Rui Mendes, José P Pinto, Eugénio C Ferreira, Jens Nielsen, Kiran Raosaheb Patil, Isabel Rocha

**Affiliations:** 1 Department of Informatics/CCTC, University of Minho, Campus de Gualtar, 4710-057 Braga, Portugal; 2 IBB-Institute for Biotechnology and Bioengineering/Centre of Biological Engineering, Universidade do Minho, 4710-057 Campus de Gualtar, Braga, Portugal; 3 Center for Microbial Biotechnology, Department of Systems Biology, Building 223, Technical University of Denmark, DK-2800 Kgs. Lyngby, Denmark; 4 Systems Biology, Dept. Chemical and Biological Engineering, Chalmers University of Technology, Kemivägen 10, SE-412 96, Gothenburg, Sweden

## Abstract

**Background:**

One of the greatest challenges in Metabolic Engineering is to develop quantitative models and algorithms to identify a set of genetic manipulations that will result in a microbial strain with a desirable metabolic phenotype which typically means having a high yield/productivity. This challenge is not only due to the inherent complexity of the metabolic and regulatory networks, but also to the lack of appropriate modelling and optimization tools. To this end, Evolutionary Algorithms (EAs) have been proposed for *in silico *metabolic engineering, for example, to identify sets of gene deletions towards maximization of a desired physiological objective function. In this approach, each mutant strain is evaluated by resorting to the simulation of its phenotype using the Flux-Balance Analysis (FBA) approach, together with the premise that microorganisms have maximized their growth along natural evolution.

**Results:**

This work reports on improved EAs, as well as novel Simulated Annealing (SA) algorithms to address the task of *in silico *metabolic engineering. Both approaches use a variable size set-based representation, thereby allowing the automatic finding of the best number of gene deletions necessary for achieving a given productivity goal. The work presents extensive computational experiments, involving four case studies that consider the production of succinic and lactic acid as the targets, by using *S. cerevisiae *and *E. coli *as model organisms. The proposed algorithms are able to reach optimal/near-optimal solutions regarding the production of the desired compounds and presenting low variability among the several runs.

**Conclusion:**

The results show that the proposed SA and EA both perform well in the optimization task. A comparison between them is favourable to the SA in terms of consistency in obtaining optimal solutions and faster convergence. In both cases, the use of variable size representations allows the automatic discovery of the approximate number of gene deletions, without compromising the optimality of the solutions.

## Background

Increasing necessity for sustainable manufacturing processes is driving a trend to replace the traditional methods of chemical synthesis by biotechnological approaches, in order to produce a number of valuable products, such as pharmaceuticals, fuels and food ingredients. This, however, implies that the microorganisms' metabolism usually needs to be modified to comply with industrial purposes, rather then to follow their natural aims like, for example, the maximization of biomass growth.

In the last few years, within the field of Metabolic Engineering, a number of tools have been developed in order to introduce genetic modifications capable of achieving the production of the desired products [1,2]. However, these have still been based mostly on qualitative or intuitive design principles and scarcely on effective mathematical models that can accurately predict cellular behaviour.

A number of attempts have been made to model the whole cell behaviour [3], but these models are still incomplete due to the lack of kinetic and regulatory information.

Nevertheless, it is possible to predict cellular metabolism, under some assumptions, namely considering steady-state conditions and imposing a number of constraints over the rates of reactions.

This is the way followed by the Flux Balance Analysis (FBA) approach [4], where the flux over a particular reaction is typically optimised using linear programming, resulting in a value for the fluxes of all reactions in the cell. The most usual approach, under this framework, is to define a reaction for biomass production and to consider this as the objective function, thus assuming that the microbes have evolved towards optimal growth [5].

Using this technique, it is possible to predict the behaviour of a microorganism, both in its wild type and mutant forms, under a number of environmental conditions. A bi-level optimization problem can then be formulated, by adding a layer that searches for the best mutant that can be obtained by simply deleting a few genes from the wild type. The idea is to force the microorganisms to produce the desired product by selected gene deletions. Therefore, the underlying optimization problem consists in reaching an optimal subset of gene deletions to maximize an objective function related with the production of a given compound.

A first approach to tackle this problem was the *OptKnock *algorithm [6], where mixed integer linear programming (MILP) is used to reach an optimum solution. An alternative approach was proposed by the *OptGene *algorithm [7] that considers the application of Evolutionary Algorithms (EAs) in this context. Since EAs are a meta-heuristic optimization method, they are capable of providing solutions in a reasonable amount of time, although this solution might not be the optimal one. Still its application in the context of the yeast *S. cerevisiae *allowed the optimization of an industrially important non-linear objective function related with productivity in several processes such as the production of succinic acid or vanillin.

*OptGene *proposed EAs with two alternative solution representation schemes: binary or integer. The binary representation is closer to the natural evolution of microbial genomes, but is more complex and leads to solutions with a larger number of knockouts. The integer representation allowed for a more compact genome in the EA, encoding only for the gene deletions. However, one of the major limitations of this representation in *OptGene *is the need to define *a priori *the number of gene knockouts, a parameter that remains fixed throughout the EA's evolution.

In this work, the authors propose a set-based representation that considers a variant with variable-sized solutions (sets of genes). This allows for the consideration of solutions with a different number of knockouts (gene deletions) during the optimization process, avoiding the trial and error approach for determining the optimum number of knockouts in a particular problem.

Under this new framework, two optimization algorithms were developed: Simulated Annealing (SA) and Set-based Evolutionary Algorithms (SEAs). Both search for the optimum set size in parallel with the search for the optimum set of gene deletions.

Although the proof of principle of the applicability of meta-heuristics to the problem of microbial strain design has already been achieved [7], a thorough validation based on the collection of sufficient data to perform statistical analysis was needed.

Therefore, in this paper, we present the results obtained by the application of the two novel methodologies to four case studies where *S. cerevisiae *and *E. coli *are the target microorganisms. In these cases, the objective function is related to the production of succinic and lactic acid, respectively. In the *in silico *experiments, the proposed SA and SEAs, and also variants with fixed size solutions were compared.

For each experiment, the algorithms were run 30 times allowing a sufficient number of function evaluations in each run, and the results obtained allowed not only to perform statistical analysis and a valid comparison between the approaches, but also to obtain a close to optimum family of solutions that were analyzed resembling their biological significance.

## Results

### Solution representation and evaluation

The optimization problem addressed in this work consists in selecting, from a set of genes in a microbe's genome, a subset to be deleted in order to maximize a given objective function, related to the microorganism's metabolism. The first issue to address, when developing an algorithm to tackle this task, is the encoding of the solutions.

In this work, a novel set-based representation is proposed, where only gene deletions are represented in the solution. Each solution consists of a set of integer values representing the genes that will be knocked out. Therefore, if the set contains the value *i*, this means that the gene(s) corresponding to the *i*-th reaction in the microbe's metabolic model will be deleted. Each element of the set is, therefore, an integer with a value between 1 and the total number of reactions, *N*, and no repeated elements are allowed. Two variants of this representation can be defined, considering either fixed or variable sized sets.

The solutions are evaluated by taking all values in the set, and forcing the fluxes of the reactions encoded by those genes to the value 0, thus adding new constraints to the metabolic model. The process proceeds with the simulation of the mutant. In this work, this is achieved using FBA (see the Methods section) but other methods can be considered at this stage (e.g. MOMA [8]). The output of this step is the set of values for the fluxes over all the reactions, some of which are then used to compute the fitness value, given by an appropriate objective or fitness function.

In this work, the adopted fitness function is the Biomass-Product Coupled Yield (BPCY) [7], given by:

$$BPCY=\;\frac{PG}{S}$$

where *P *stands for the flux representing the excreted product; *G *for the organism's growth rate (biomass flux) and *S *for the substrate intake flux.

**Figure 1:**
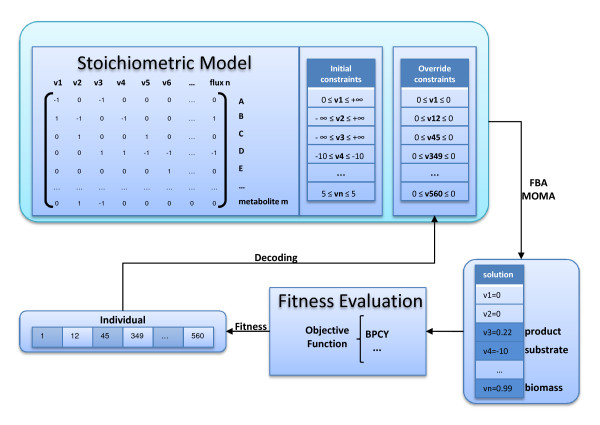
**The process of solution decoding and evaluation**. The solutions (individuals) are encoded using the proposed set-based representation where the genes to be deleted are represented. Each individual is decoded by imposing additional constraints to the original metabolic model. FBA is the method used to simulate the cellular behaviour that will be associated to a given fitness, related with the productivity in a given interesting compound.

Besides optimising for the production of the desired product, this objective function also allows to select for mutants that exhibit high growth rates, i.e., that are likely to exhibit a high productivity, an important industrial aim. The overall process of decoding and evaluating a solution is depicted in Figure 1.

### Evolutionary Algorithms

Evolutionary Algorithms (EAs) [9] are a popular family of optimization methods, inspired in the biological evolution through natural selection. These methods work by evolving a population, i.e. a set of individuals that encode solutions to a target problem in an artificial chromosome. Each individual is evaluated through a fitness function that assigns it a numerical value, corresponding to the quality of the encoded solution. New individuals (solutions) are created by the application of reproduction operators to selected parents and, since the pool of parents is taken from the previous population using probabilities, EAs are stochastic in nature.

The proposed set-based EA (SEA) uses the set-based representation and defines two reproduction operators: a crossover and a mutation. The crossover operator is inspired on traditional uniform crossover operators [10] and works as follows: the genes that are present in both parent sets are kept in both offspring; the genes that are present in only one of the parents are sent to one of the offspring, selected randomly with equal probabilities. The mutation operator is a random mutation that replaces an element of the set by another, randomly generated in the allowed range (1 to N).

In SEAs, a minimum and a maximum value for the set size are defined. If these values are equal, the search only goes through sets of a given cardinality. The operators comply with this constraint by creating solutions always of the same size. In the case of the crossover, this implies that, when selecting the destination of the genes that are present in only one parent, if an offspring reaches the maximum number of elements in the set, the remaining genes go to the other offspring.

If the maximum and minimum values of the set sizes are different, variable-sized sets can be encoded and compete within the same population. In this case, two additional mutation operators are defined in order to create solutions with a distinct size:

• *Grow*: consists in the introduction of a number of new elements into the set, whose values are randomly generated in the available range, avoiding duplicates.

• *Shrink*: a number of randomly selected elements are removed from the set.

In both cases the limits on the set size are strictly obeyed. The Grow and Shrink mutation operators are each used with a probability of 5% each, meaning that 10% of the new individuals are created in this way. The remaining ones are bred by the aforementioned crossover and mutation operators with equal probabilities. In the experiments reported in this work, when a variable size SEA is used, the minimum size is set to 1 and the maximum size is set to N, thus not restricting the possible range of solutions.

SEA uses a selection procedure that consists in converting the fitness value into a linear ranking of the individuals in the population, and then applying a roulette wheel [11] scheme. In each generation, 50% of the individuals are kept from the previous generation, and 50% are bred by the application of the reproduction operators. An elitism value of 1 is used, allowing the best individual of the population to be always kept.

An initial population is randomly created and the termination criterion is based on a fixed number of generations (in this work this is calculated to achieve a given maximum number of solution evaluations). In the variable size SEAs, the size of the sets encoded in the initial individuals is randomly set to a value between 1 and 10.

### Simulated Annealing

Simulated Annealing (SA) [12] is an optimization algorithm where a single solution evolves by successive small changes (mutations) to achieve an approximation to the global optimum. Better solutions are always accepted and local optima are avoided by the fact that SA allows worse solutions to replace the current one with a certain probability that decreases over time. This probability is controlled by the value of a parameter, denoted as temperature given the fact that SA is loosely inspired by the annealing process used in many different areas (e.g. in metallurgy or PCR reactions) where the system initially at a high temperature, is slowly cooled so that the system at any time is approximately in thermodynamic equilibrium.

In optimization, the current state is a solution to the problem and its fitness value states for the system's energy. The current solution is represented using similar encoding schemes as the ones used in the EAs. This allows SA to be applied for instance to problems with binary, integer or real variables. In this work, we developed an SA with the set-based representation previously explained in the context of the SEA.

At each step, the algorithm works by creating a new solution from the current one, using mutation operators. The SA variant developed in this work allows the use of a combination of mutation operators, similar to the ones described for the EAs, each with a given probability. When a new solution is created and evaluated, the difference between the previous and the new fitness values is computed (Δ*E*). A better solution is always accepted, while a worse one is only accepted with a probability given by the Boltzmann factor:

$$p[accept]=e^{-\frac{\Delta E}{T}}$$

The temperature *T *is initialized to T_0 _and it is decreased according to a given cooling schedule that represents how this value decreases along the algorithm. The entire process is repeated until the temperature is sufficiently low. For each temperature value, a number of iterations are performed, sufficient to give a good sampling statistics.

The main configuration parameters of the SA are the initial and final temperatures, the number of iterations performed at each temperature and the cooling schedule used. The choice of these parameters is of paramount importance to the performance of the algorithm. If the initial temperature is too low or the cooling schedule is not slow enough, the optimization process may become stuck in a local optimum. On the other hand, if the initial temperature is too high, the cooling is too slow or the number of iterations per temperature is too high, the algorithm wastes a potentially large amount of computational time searching for solutions.

The cooling schedule used in this work is among the most popular ones, where the temperature decreases exponentially, according to the following equation:

*T*_*n*+1 _= α*T*_*n*_

where α is a scale parameter (0 < α < 1).

To ensure that the cooling schedule is sufficiently slow, the parameter α should be given values close to the unity. The choice of initial (*T*_0_) and final temperatures (*T*_*f*_) is problem dependent and its definition poses serious problems. Indeed, it is easier to think in terms of the objective function values (fitness landscape) than in terms of values for the temperature. Thus, the following auxiliary parameters were defined:

• Δ*E*_0 _– The difference in energy that corresponds to an acceptance probability of worse solutions of 50%, at the beginning of the run;

• Δ*E*_*f *_– The difference in energy that corresponds to an acceptance probability of worse solutions of 50%, at the end of the run;

• *trials *– The number of iterations per distinct temperature value;

• *NFEs *– The number of function evaluations.

Using these parameters, the initial temperature, the final temperature and the scale parameter were computed using the following equations:

$$\begin{array}{l} T_{0}=-\frac{\Delta E_{0}}{\log0.5}\\ T_{f}=-\frac{\Delta E_{f}}{\log0.5}\\ \alpha =\exp\left(\frac{\log T_{f}-\log T_{0}}{\left(\frac{NFEs}{trials}\right)}\right) \end{array}$$

The advantage of using Δ*E*_0 _and Δ*E*_*f *_is that it allows the user who approximately knows the fitness landscape of the problem to automatically define the temperatures by reasoning over the values of the objective function. Furthermore, by supplying the number of function evaluations instead of the scale parameter α enables the comparison with other optimization approaches.

As in the EA, two variants of this representation can be defined, considering fixed or variable sized sets. In the fixed-size alternative, the previously defined random mutation operator is used. In variable-sized representations, the two additional mutation operators (Grow and Shrink) are also used, each with a probability of 25%, meaning that half of the new individuals are created in this way.

In brief, the SA algorithm searches for the optimal set of gene deletions by exploring the whole search space (all combinations of gene knockouts) in a stochastic manner, where the probability of accepting a non-optimal search direction is high in the beginning and very low or zero in the end. The probability of accepting a non-optimal direction allows the algorithm to avoid the local optimal solutions. Thus, the algorithm can find combinations of gene deletions which individually may not necessarily lead to the improved production.

**Figure 2:**
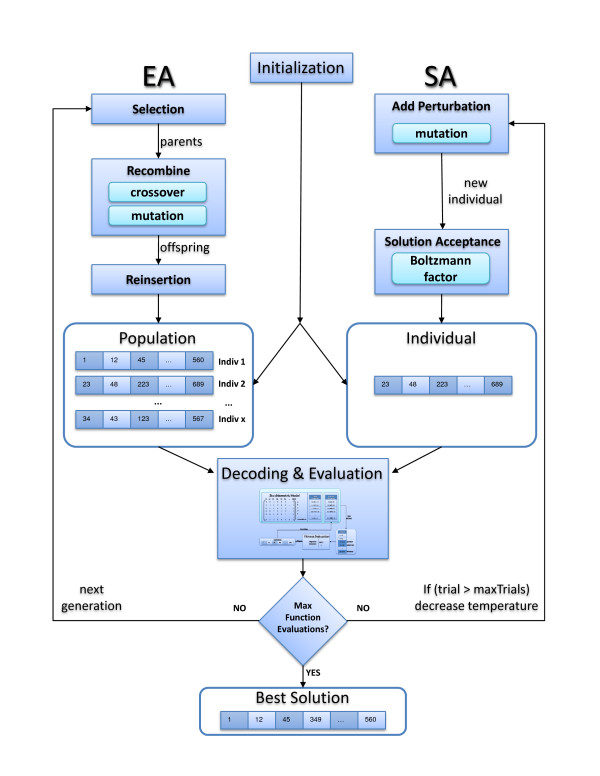
**Comparison of the EA and SA algorithms**. A scheme illustrating the major steps of the EA and SA algorithms developed in this work. Each individual represents a set of genes/reactions to be deleted from the model. The task is to find one or more individuals that are predicted to have high yield or productivity (in general, any flux phenotype). The prediction of the flux phenotype is based on optimality principles of metabolic networks such as Flux Balance Analysis.

An overview of the major steps in the SEA and SA algorithms is provided in Figure 2.

### Greedy algorithm

A greedy algorithm was devised to provide a baseline comparison with the more elaborate optimization approaches. This algorithm tries to explore the search space efficiently, by combining local search (the exploration of the neighbourhood of known solutions) and exhaustive search. The exhaustive search starts with the wild type and proceeds with an increasing number of knockouts. When a solution that improves over the wild type one is found, local search is used to recursively improve it by adding knockouts. When no further improvements can be obtained with this local search procedure, exhaustive search is resumed. The details on the implementation of this algorithm are given in the Methods section.

### Pre-processing and post-processing

Typically, in microbial genome-scale models, the number of variables (fluxes over metabolic reactions) is quite high (hundreds or even a few thousands) and therefore the search space is very hard to address by the optimization algorithms. Thus, every operation that gives a contribution to reduce this number, without compromising the quality of the solutions, greatly improves the convergence of the methods used.

In this work, a number of operations were implemented in order to reduce the search space, being described in the Methods section.

### Case studies

Four case studies were used to test the aforementioned algorithms. The first considers *S. cerevisiae *and the aim is to produce succinic acid, while the remaining considers *E. coli *and the production of lactic acid (in aerobic and anaerobic conditions) and succinic acid. All use glucose as the main substrate.

Succinic acid is a chemical used as feedstock for the synthesis of a wide range of other chemicals with several industrial applications. Succinic acid and its derivatives have been used as common chemicals to synthesize polymers, as additives and flavouring agents in foods, supplements for pharmaceuticals, or surfactants. Currently, it is mostly produced through petrochemical processes that can be expensive and have significant environmental impacts. Succinic acid, therefore, represents an important case study for identifying metabolic engineering strategies [13]. In fact, the knockout solutions that lead to an improved phenotype regarding the production of succinic acid are not straightforward to identify since they involve a considerable number of interacting reactions.

Lactic acid and its derivatives have been used in a wide range of food-processing and industrial applications like meat preservation, cosmetics, oral and health care products and baked goods. Additionally, as lactate can be easily converted to readily biodegradable polyesters, it is emerging as a potential material for producing environmentally friendly plastics from sugars [14].

Several microorganisms have been used to commercially produce lactic acid [15], such as *Lactobacillus *strains. However, those bacteria also have undesirable traits, such as a requirement for amino acids and vitamins which complicates acid recovery. *E. coli *has many advantageous characteristics as a production host, such as rapid growth under aerobic and anaerobic conditions and simple nutritional requirements. Moreover, well-established protocols for genetic manipulation and a large knowledge on this microbe's physiology enable the development of *E. coli *as a host for production of optically pure D- or L-lactate by metabolic engineering [16,17].

Although reported work have been focused on the anaerobic production of lactic acid, it is in principle possible to develop aerobic processes, since the carbon overflow in *E. coli *towards acetic acid in aerobic conditions can be diverted to the production of lactate. The main advantage of such a process is that, since *E. coli *reproduces much faster in aerobic conditions, it should be possible to improve the productivities when compared with anaerobic processes.

The genome-scale stoichiometric models used for *S. cerevisiae *and *E. coli *were developed by [18,19]. The details of each model are given in Table 1.

**Table 1:** Statistics of the genome-scale models used in the case studies

	***S. cerevisiae***	***E. coli***
Number of fluxes	1104	1075

Number of metabolites	825	761

Number of fluxes after pre-processing	460	549

Number of variables in the optimization	268	301

The details on the original models are given, together with the statistics of the models after the pre-processing steps.

### Experiments

A systematic set of experiments was conducted to evaluate the performance of the proposed SEA and SA algorithms. These were applied to the four case studies, using both their fixed and variable size variants. In the fixed-size case, several alternatives for the cardinality of the set (*k*) were considered, being used the following values for *k*: 2, 4, 6, 8, 10, 12 and 20. The experimental setup is given in the Methods section.

The full set of results can be found in the additional files 1 to 3, including a set of statistics calculated over the 30 runs performed in each scenario, the set of knockouts in the overall best solutions and also the frequencies of occurrence of each gene deletion in the results of each optimization scenario. The analysis of these results will be conducted in the next section.

## Discussion

### Reaching the optimum solution size

**Figure 3:**
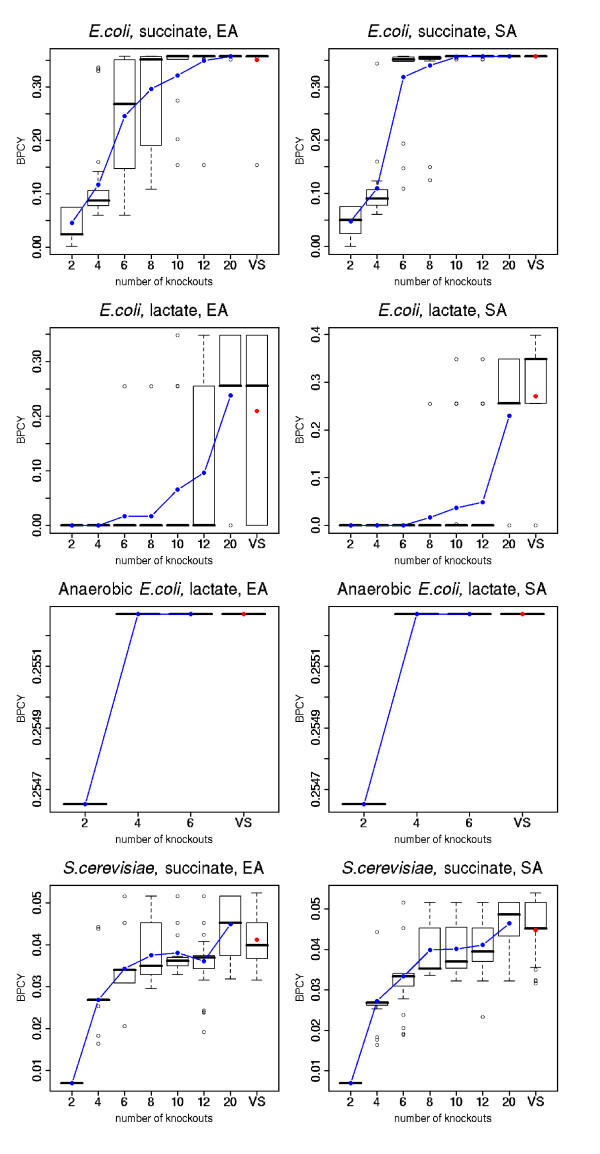
**Boxplots with the results obtained by the SA and SEA**. The eight graphs (one for each case study and algorithm SA or SEA) show a set of boxplots (one for each value of the k, the number of knockouts, and one for the variable size variant) with the following statistics: best value (maximum), quartiles, median and minimum value; the mean value is also shown as a blue dot (or a red dot in the case of the variable size). All values are calculated over the 30 runs for each scenario.

In Figure 3, a summary of the main statistics regarding the objective function (i. e. best result, mean, median and quartiles over the 30 runs) obtained for both algorithms (fixed and variable size variants) is plotted. The plots make clear the improvement of the results when the value of *k *increases (higher means and lower variability), until an optimum level is reached.

The variable size alternatives are normally able to find results of the same quality of the best values of *k*, therefore being able to automatically discover good values for this parameter. Therefore, the use of the variable size alternative allows the user to save a considerable amount of time in computation.

Also, one important feature in this problem is the ability to find good solutions with the minimum number of gene deletions, since this will make easier its implementation in the lab. In Table 2, the average size of the best solutions found by the variable size variants of the SEA/SA are shown. It is important to note that the size of the solutions for each algorithm is computed from the best solutions found in each run that undergo a simplification process (explained in the Methods section, post processing methods).

**Table 2:** Size of the best solutions obtained by the variable size SEA/SA

**CASE STUDY**	**SEA**	**SA**
*S. cerevisiae*, succinate, aerobic	38	17

*E. coli*, lactate, aerobic	8.7	9

*E. coli*, lactate, anaerobic	3	3.6

*E. coli*, succinate, aerobic	15	16

This table shows the average number of knockouts in the solutions obtained by the variable size variants of SEA and SA. Only the best solutions found over the 30 runs are considered.

From the values on this table and the results shown in Figure 3, it is clear that the variable size variants do not return solutions with very large sets of knockouts, when compared to the fixed size approaches. Indeed, it seems that these solutions only "grow" during the evolution in SEA/SA if the new knockouts provide fitter solutions.

### Comparison between the performance of the algorithms

To provide a baseline result for the comparison between the algorithms, the greedy algorithm described before was applied to the four case studies. The termination criterion was to perform 5 million function evaluations (100 times the value used in the SEA/SA). The results are given in Table 3.

**Table 3:** Results obtained by the greedy algorithm for the case studies

**CASE STUDY**	**BPCY of best solution**	**Number of knockouts**
*S. cerevisiae*, succinate, aerobic	0.03260	23

*E. coli*, lactate, aerobic	0.00000	-

*E. coli*, lactate, anaerobic	0.25527	3

*E. coli*, succinate, aerobic	0.07779	3

This table shows the results obtained by the greedy algorithm. The first column shows the details on the case study (organism, desired product, conditions); the second column states the value of the objective function (BPCY) and the last column the number of knockouts, both obtained for the best solution found.

The results confirm that the optimization problems are quite difficult to solve, since in most case studies the greedy algorithm cannot find good results. The exception is the *E. coli*, lactate, anaerobic case study that seems an easier task. The difficulty of the case studies is also visible in the small number of runs where the best solutions are found, both by the SEA and the SA (see additional file 1). This fact leads to an important conclusion regarding the use of these stochastic methods: it is normally necessary to run SEA or SA multiple times to guarantee that a good solution is achieved.

Regarding the comparison of the SEA/SA with the greedy algorithm, both the SEA and SA perform a quite efficient exploration of the search space, since although conducting only 1% of the number of solution evaluations, they are able to obtain much better results.

Comparing the performance of the SEA and SA algorithms, they seem to be at a very similar level, in most cases with overlapping confidence intervals. When comparing the variable size variants of both algorithms, the SA seems to be more reliable, showing good results in all case studies and smaller variability across the 30 runs.

Two additional features that are important when comparing meta-heuristic optimization algorithms are the computational effort required and the convergence of the algorithm to a good solution. The computational burden of the alternatives compared (SEA and SA) is approximately the same, since the major computational effort is devoted to fitness evaluation and the same number of solutions is evaluated in each case. A typical run of each algorithm for the case studies presented will take approximately one hour in a regular PC.

**Figure 4:**
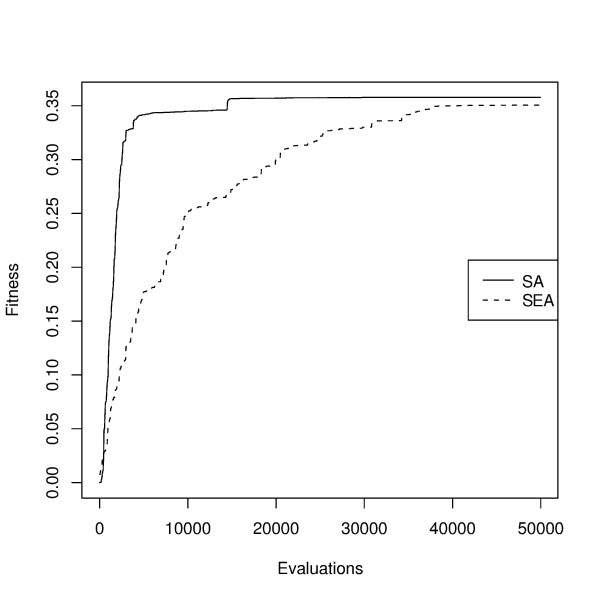
**Convergence plot of SA and SEA**. Convergence plot of the SA and SEA algorithms (variable size variant) in the case study with *E. coli *production of succinate (x-axis represents the number of function evaluations; y-axis plots BPCY values)

Regarding the convergence of the algorithms, a plot of the evolution of the objective function along the generations of the SA and SEA is given in Figure 4. The case study regarding *E. coli *production of succinic acid is taken as an illustrative example and the variable size variants were selected. It is clear from this plot that the SA converges faster than the SEA, obtaining high quality results earlier in the runs. This is the case also in the remaining case studies, although the results are not shown. Thus, SA allows a reduction in the computation time needed to achieve a useful solution.

**Figure 5:**
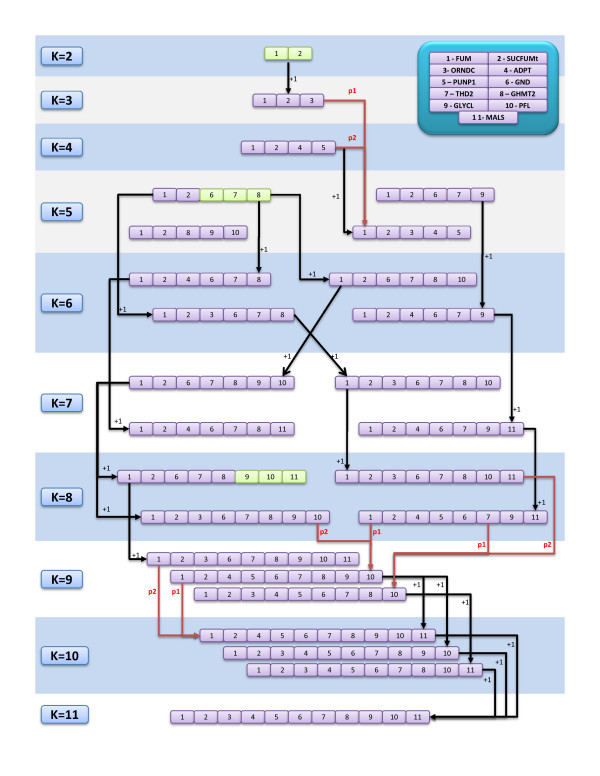
**Analysis of the best solution and partial solutions for the *E. coli*, succinate case study**. One of the best solutions found for the *E. coli*, succinate case study was analyzed. All sub-solutions, i.e. solutions with a sub-set of the original gene deletions, were evaluated. The best ones for each set size are shown in the figure. Possible ways to reach a solution are shown (in black, grow mutations; in red, crossover operations).

An additional analysis was performed in order to better understand the reasons why the SA seems to perform better. The first was a study of one of the best solutions found for the *E. coli*, succinate case study and its partial solutions (solutions with a subset of the knockouts included in the original solution). The aim was to understand how the algorithms build the final solution from smaller ones, along evolution, using mutation and/or crossover operators. The result of this analysis is displayed in Figure 5.

A look into the figure shows that in most cases the solutions with more knockouts can be obtained by adding one or two knockouts to smaller solutions. This leads to the insight that the crossover operator is probably redundant in this process. To test this hypothesis, an EA only with mutation operators was tested (only the variable size variant was tested in this study). The results for this algorithm are given in additional file 4, and its analysis shows that they are quite similar to the ones obtained by the SEA with crossover. Thus, the crossover operator does not seem to be profitable in terms of the EA's performance, at least in these case studies. This is probably due to the lack of building blocks (subsets of two or more knockouts that are associated with a good fitness) that can be combined to achieve better solutions. To study if there are case studies where this could happen remains as interesting further work.

It should be noticed that, although EAs have performed worse, their application in this field should still be considered as, when compared with SA, they allow an easier parallelization of the computation given their population based nature. This represents an important advantage when algorithms with heavy computational demands are compared.

### Analysis of the solutions

A thorough analysis of the solutions obtained is out of the scope of this paper, but it should be pointed out that some of the knockouts obtained are unlikely to have a biological meaning (with a view point of either *in vivo *implementation or viability of the resulting strains). The very large number of knockouts required for the best solutions for succinate production in both yeast and *E. coli *are un-realistic to realize in reality (see Table 4 and Supplementary File 2).

**Table 4:** Best overall mutants obtained for each case study

**CASE STUDY**	**BPCY**	**List of knockouts**
*S. cerevisiae*, succinate, aerobic	0.05398	PGI1_1, PGI1_2, FBP1, PDC6, ADH4, SDH3_2, AAH1_1, URH1_1, U30_, MET3, ALD4_2, GSH1, U103_, YER053C, CTP1_1*

*E. coli*, lactate, aerobic	0.39850	ALCD19, DRPA, GLYCDx, F6PA, TPI, LDH_D2, EDA, TKT2, LDH_D-

*E. coli*, lactate, anaerobic	0.25527	FRD3, GART, ADHEr *

*E. coli*, succinate, aerobic	0.35785	MALS, ORNDC, FUM, GLYCL, GHMT2, ADPT, DCYTD, DUTPDP, URIDK2r, NTD8, PUNP1, THD2, GND, PFL, SUCFUMt *

This table shows the list of knockouts in the best solution found in all the runs of both algorithms. In the rows marked with *, other solutions with the same BPCT exist; the one with less knockouts was selected (see Additional file 2 for the complete list)

Furthermore, some of the knockouts obtained are related with fluxes associated with transport reactions that are not necessarily enzyme catalyzed or belong for example to nucleotide pathways. Although these reactions are not included in the set of essential genes validated experimentally [19], it is possible that the removal of the corresponding genes will originate non-viable mutants or that the mutation will not contribute to increase the production of the target compound. For most of the cases, however, even *in silico*, such mutations contribute very slightly to the objective function value. These results emphasise the need of analysing the solutions in the context of additional biological information before lab implementation. Nevertheless, analysis of the connection between these mutations and the objective function may help to gain insight into the bottlenecks for the production of the desired compounds.

Interestingly, if the solutions obtained are compared with real implementation of knockout mutants, as is the case of succinate production with *E. coli*, for which there are several works published, for example [20], where a penta-mutant is described, the best solutions obtained by the algorithms are quite different from the ones implemented, although in some cases the reactions to be eliminated belong to the same pathways. Comparing our results with the work described in [20], the penta-mutant described there was not reached by our algorithms, a situation that is easily explained by the fact that one of the genes deleted (icd) is *de facto *an essential gene in the stoichiometric model of *E. coli *used, since its deletion leads to zero growth. Furthermore, the simulation of the tetra-mutant obtained (excluding icd) only produces succinate *in silico *if the oxygen uptake is constrained, which is consistent with what is described in [20] regarding pyruvate accumulation for this mutant. In fact, the strategy used in [20] is partly based on diverting the overflow in glycolysis from acetate to succinate. Since the stoichiometric model does not accurately predict the overflow phenomenon in *E. coli*, it is difficult to obtain such strategies using our algorithms. This analysis emphasises the importance of using reliable stoichiometric models with these algorithms. Although for *E. coli *the model used has been validated for many different situations, there are clearly still some discrepancies between simulation results and reality, namely concerning essential genes. It would be interesting to know if the mutants obtained *in silico *based on our approaches perform better or worse than the ones based on empirical knowledge of the metabolic pathways. Some of the obtained knockouts point to changes in the citrate cycle, either by the deletion of succinate dehydrogenase or fumarase, which is also consistent with some of the approaches used in [20]. In the case of yeast, the suggested strategies also include deletion of succinate dehydrogenase, which is the main succinate consuming reaction under aerobic conditions. Since yeast can grow without flux through this reaction, the coupling of the objective function (succinate production) to the growth is achieved by suggesting the deletion of other pathways. For example, deletion of THR1 necessitates production of Threonine (an essential amino acid) *via *the glyoxylate cycle where Succinic acid is produced.

Looking again at additional file 1, the fact that among the 30 runs multiple solutions are found with close to optimum objective function values is an interesting feature, meaning that, especially for "difficult" case studies, there are many combinations of knockouts that give good solutions. More generally, it has been experimentally shown that many different and non-intuitive combinations of genetic modifications can lead to product enhancement (for example, see [21]). Many of the effects of genetic changes on the desired objective function are due to kinetic and regulatory effects apart from the stoichiometry which is the area of focus in this study.

Interestingly, our results point out that even stoichiometric model leads to several distinct solutions. This variety is due to the large possible operating space of the cellular metabolic pathways [22,23]. The number of alternative solutions will thus be a function of the number of elementary flux modes that span the desired range of design and biological objective functions. Since, in general, it is difficult to account for the kinetic and regulatory information in the genome-scale models, the variety of the solutions generated from our approach can serve as a compendium of hypotheses that can be subsequently manually screened based on the available regulatory and kinetic data about the systems under investigation. Regulatory and kinetic constraints are difficult to explicitly incorporate into the genome-scale models due to their non-linear nature and lack of reliable estimates of *in vivo *kinetic parameters and metabolite concentrations. The productivity and flux data available in literature for certain mutants can nevertheless be used to impose additional constraints on the models. The ability of the here proposed algorithms to rapidly and effectively search large solutions space provide us new opportunities to handle more complex problems where some of the available regulatory information can be incorporated into the fitness function.

We also here note that the strategies generated by our algorithm inherently exploit the robustness of the network in face of multiple knock-outs [24]. The alternative strategies (including non-optimal strategies) can be classified into two broad categories: i) strategies where two sets of deletion targets that are interchangeable due to their close (or identical) biochemical relation, and, ii) biochemically different strategies leading to similar objective function values.

One clear example from the first category is SDH3_1 and SDH3_2 targets found for the improvement of succinate productivity in yeast. These two are biochemically coupled reactions. In fact, both of these reactions are catalyzed by the same Sdh3 protein complex. Similar example is two reactions in the respiratory chain, FRD3 (fumarate reductase) and NADH8 (NADH dehydrogenase) which were found as part of two different deletion strategies for the improvement of lactate in *E. coli *under anaerobic conditions. For the same objective function under aerobic conditions, acetate kinase (ACKr) and phosphotransacetylase (PTAr) distinguishes two solutions obtained for 6 deletion search. The product of both of these reactions is Acetyl phosphate, albeit obtained *via *different substrates.

One example of the second category (biochemically different strategies that lead to the desired product formation) is illustrated in two proposed gene deletion sets: [SUCD4, ENO, PGK, HSK] and [SUCD4, GHMT2, THD2, GND] identified in the Succinic acid case study for *E. coli*. Apart from SUCD4 (succinate dehydrogenase) the rest of the genes span different parts of metabolism: Glycine and serine metabolism, Oxidative phosphorylation and Pentose phosphate cycle in the first set; and Threonine and lysine metabolism and Glycolysis in the second set.

## Conclusion

The development of efficient and accurate modelling and optimization methods in Metabolic Engineering has a considerable impact in Biotechnology, leading to substantial economical gains in areas such as the production of pharmaceuticals, fuels and food ingredients.

In this work, a contribution to this arena was provided by the development of Evolutionary Algorithms and Simulated Annealing that are able of reaching near optimal sets of gene deletions in a microbial strain, in order to maximize the production of a given product. An important novel feature of this work was the introduction of set-based representations that made use of variable size sets of gene deletions. This allows the automatic definition of the optimum number of gene deletions, in parallel with the search for the best knockouts.

A systematic statistical validation of the algorithms was conducted, where those were tested, in several variants, in four case studies that dealt with the production of succinic and lactic acid by the bacterium *E. coli *and the yeast *S. cerevisiae*.

A number of features can be introduced and/or improved in this work. These include other algorithms for simulation and distinct objective functions. Regarding the former, an alternative algorithm for simulating mutants' phenotype is the MOMA algorithm that was proposed by [8], where it is assumed that knockout metabolic fluxes undergo a minimal redistribution with respect to the flux configuration of the wild type. It would also be very interesting to consider an objective function capable of taking into account the number of knockouts of a given solution and the cost of its experimental implementation.

One other area of future work is the development of multi-objective optimization algorithms that are able to provide in a single run, not only a single solution but rather a whole set of distinct trade-offs between the two goals: maximizing biomass and maximizing the desired product.

## Methods

### Flux Balance Analysis

The Flux Balance Analysis approach is based on a steady state approximation to concentrations of the internal metabolites, which reduces the corresponding mass balances to a set of linear homogeneous equations. For a network of M metabolites and N reactions, this is expressed as:

$$\begin{array}{cc} \sum_{j=1}^{N}S_{ij}v_{j}=0, & i=1,....M \end{array}$$

where *v*_*j *_corresponds to the rate of reaction *j*, or to the *j*^*th *^metabolic flux and the stoichiometric coefficient, *S*_*ij*_, stands for stoichiometric coefficient of metabolite i in reaction *j*.

For most metabolic networks, the number of fluxes is greater than the number of mass balance constraints, resulting in an underdetermined system.

Besides these stoichiometric constraints, thermodynamic and capacity constraints can be added as inequalities:

α_*j *_≤ ν_*j *_≤ β_*j*_,     *i *= 1, .... *M*

FBA allows the detailed examination of the model via the use of linear programming to determine the optimal flux distributions using a specified linear objective function:

$$\begin{array}{l} \text{Maximize Z}\\ \begin{array}{cc} \text{Subject to} & \begin{array}{cc} \sum_{j=1}^{N}S_{ij}v_{j}=0, & i=1,...M \end{array}\\ & \begin{array}{cc} \alpha_{j}\leq v_{j}\leq \beta_{j}, & j=1,...N \end{array} \end{array} \end{array}$$

For metabolic applications, the linear objective function (Z) to be maximized or minimized can correspond to different objectives ranging from a particular metabolic engineering design objective (for example, optimization of a metabolite production) to the maximization of cellular growth. Since studies in several different organisms have demonstrated that their metabolic networks have evolved for the optimization of the specific growth rate under several carbon source-limiting conditions, the most commonly used objective function is the maximization of the biomass formation reaction rate.

### Greedy algorithm: detailed description

This algorithm is based on the evaluation of a pre-defined maximum number of solutions that are obtained in the neighbourhood of the best ones found and by using exhaustive search when no local search can be performed. The main steps are the following:

1. A list L of solutions to explore is created, initially containing one single element: the wild type solution, i.e. a solution with an empty set of knockouts.

2. While the maximum number of solutions has not been evaluated, one of the following steps is performed:

*2.1. Local search*. If L is not empty, the solution *s *in its head is removed from L. All solutions in the neighbourhood of *s *are explored by local search (the neighbourhood of a solution is the set of all possible solutions obtained by adding one gene deletion to the original set of knockouts). The solutions that improve over the original one are added to the list such that their neighbourhoods can be further explored.

*2.2. Exhaustive search*. If L is empty, the next solution from an exhaustive search process is taken. Solutions are obtained starting with the wild type and proceeding to all solutions with 1 knockout, 2 knockouts, and so on. If there is an improvement over the wild type solution, this solution is added to the head of the list; else the algorithm proceeds to the next solution.

### Pre-processing and post-processing methods

The pre-processing operations performed to simplify the genome-scale metabolic model were the following.

• Detection of reactions for which the fluxes, given the constraints of the linear programming problem, cannot exhibit values different from 0. For every reaction in the model, two linear programming problems are solved: the first is defined by setting the flux over that reaction as the maximization target, while for the second the same variable is minimized. If both problems have an objective function of 0, the variable is removed from the model.

• Detection of equivalent variables, i.e. pairs of fluxes that are constrained to have the same value by the linear model. These are directly identified from the S matrix coefficients. Each group of equivalent variables is replaced by a single variable.

• Discovery of essential genes that cannot be deleted from the microorganism's genome. As these genes should not be considered as targets for deletion, the search space for optimization is reduced. For each gene in the microbe's genome, a linear programming problem instance is defined, setting the corresponding flux to 0, while maximizing the biomass flux. After running the Linear Programming algorithm, if the resulting biomass flux is zero (or near zero) the gene is marked as essential. The biological meaning of this fact is that the microbe is unable to survive when the gene is absent. It should be noted that, unlike the previous ones, this process does not imply any changes in the model, but produces information that is useful for the optimization algorithms. The list of essential genes can be manually edited to include genes that are known to be essential *in vivo*, but not *in silico*.

• Identification of fluxes that are not associated with any genes, like the ones related with external metabolites and exchange fluxes that represent transport reactions. These are not allowed to be knocked out, since generally this would not have a biological meaning.

Additionally, at the end of each SA/SEA run, the best solution goes through a simplification process. This is achieved by identifying all gene deletions that contribute to the fitness of the solution, removing all deletions that keep the objective function unaltered. The aim of this step is to keep only the necessary knockouts, given that the practical implementation of a gene deletion is both time consuming and costly.

### Implementation details

The implementation of the proposed algorithms was conducted in the Java programming language by the authors. In the implementation of the FBA algorithm, the GNU linear programming package (GLPK – ) was used to run the simplex algorithm.

### Experimental setup of the SEA/SA algorithms

The population size for SEA was set to 100. In all cases, a run of SA/SEA terminated when 50000 function evaluations were performed. The SA used Δ*E*_0 _= 0.005, Δ*E*_0 _= 5E-5 and *trials *= 50. For each experimental setup the process was repeated for 30 runs and the mean and 95% confidence intervals were calculated.

## Availability

The source code of the implementation is made available in the project's web site, together with all instructions and requirements for software installation, as well as example files for a sample model and configuration files both for EA and SA algorithms.

More details:

• Project name: Natural Computation Algorithms for In Silico Metabolic Engineering

• Project home page: 

• Operating system(s): Platform independent

• Programming language: Java

• License: GNU-GPL, version 3

## Authors' contributions

MR, IR, RM, KP and JN were involved in the conception of the algorithms. MR, RM, PM and JPP were involved in the implementation of the algorithms and software tools. IR, KP and ECF proposed and prepared the case studies and validated the results. MR, IR, KP and RM helped to draft the manuscript. All authors were involved in the analysis of the results, read, reviewed and approved the final manuscript.

## Supplementary Material

Additional file 1 **The complete results of the SEA and SA for the four case studies**. For each case study the details on the organism, the target product and the conditions are given in the header. In each table, the algorithm (SA or SEA) and the maximum number of allowed knockouts k (VS stands for variable size) are given. For each configuration the mean, the confidence interval and the best value of the objective function (BPCY) obtained over the 30 runs are provided. Furthermore, the number of runs where the best solution was reached is also shown. Click here for file

Additional file 2 **The list of the best solutions found by each algorithm and configuration**. For each case study, algorithm (SA/SEA) and configuration (value of k) the fitness of the best solution and the corresponding list of knockouts is given. Alternative optimum solutions are provided when applicable. Click here for file

Additional file 3 **The complete results of the gene frequencies analysis**. For each case study, algorithm (SA/SEA) and configuration (value of k) the frequency of the presence of each particular gene knockout within the set of near-optimal is given. The set of solutions used in this case is built from the set with the best solutions from each run, keeping the ones that are within 1% of the best overall solution (over the 30 runs). A global frequency for all values of k is calculated. Click here for file

Additional file 4 **The results of EA with only mutation operators**. For each case study and algorithm (SA/SEA) the results are shown in a way similar to the ones in additional file 1. Click here for file

## References

[B1] Stephanopoulos G, Aristidou A, Nielsen J (1998). Metabolic engineering.

[B2] Nielsen J (2001). Metabolic Engineering. Applied Microbiology and Biotechnology.

[B3] Tomita M (2001). Whole-cell simulation: a grand challenge of the 21st century. Trends in Biotechnology.

[B4] Kauffman KJ, Prakash P, Edwards JS (2003). Advances in flux balance analysis. Curr Opin Biotechnol.

[B5] Ibarra RU, Edwards JS, Palsson BO (2002). *Escherichia coli *K-12 undergoes adaptive evolution to achieve *in silico *predicted optimal growth. Nature.

[B6] Burgard AP, Pharkya P, Maranas CD (2003). OptKnock: A bilevel programming framework for identifying gene knockout strategies for microbial strain optimization. Biotechnology and Bioengineering.

[B7] Patil KR, Rocha I, Forster J, Nielsen J (2005). Evolutionary programming as a platform for in silico metabolic engineering. BMC Bioinformatics.

[B8] Segre D, Vitkup D, Church GM (2002). Analysis of optimality in natural and perturbed metabolic networks. Proceedings of the National Academy of Sciences of the United States of America.

[B9] Michalewicz Z (1996). Genetic Algorithms + Data Structures = Evolution Programs.

[B10] Syswerda G Uniform crossover in Genetic Algorithms. Proc 3rd Intl Conference on Genetic Algorithms 1989.

[B11] De Jong K (1975). An analysis of the Bahavior of a Class of Genetic Adaptive Systems.

[B12] Kirkpatrick S, Gellatt CD, Vecchi MP (1983). Optimization by Simulated Annealing. Science.

[B13] Lee SY, Hong SH, Moon SY (2002). *In Silico *metabolic pathway analysis and design: succinic acid production by metabolically engineered *Escherichia coli *as an example. Genome Informatics.

[B14] Hofvendahl K, Hahn-Hagerdal B (2000). Factors affecting the fermentative lactic acid production from renewable resources. Enzyme and Microbial Technology.

[B15] John RP, Nampoothiri KM, Pandey A (2007). Fermentative production of lactic acid from biomass: an overview on process developments and future perspectives. Applied Microbiology and Biotechnology.

[B16] Chang DE, Jung HC, Rhee JS, Pan JG (1999). Homofermentative production of D- or L-lactate in metabolically engineered Escherichia coli RR1. Appl Environ Microbiol.

[B17] Zhou SD, Causey TB, Hasona A, Shanmugam KT, Ingram LO (2007). Production of optically pure D-lactic acid in mineral salts medium by metabolically engineered Escherichia coli W3110. Applied and Environmental Microbiology.

[B18] Forster J, Famili I, Fu P, Palsson BO, Nielsen J (2003). Genome-scale reconstruction of the Saccharomyces cerevisiae metabolic network. Genome Res.

[B19] Reed JL, Vo TD, Schilling CH, Palsson BO (2003). An expanded genome-scale model of *Escherichia coli *K-12 (iJR904 GSM/GPR). Genome Biology.

[B20] Lin H, Bennett GN, San KY (2005). Genetic reconstruction of the aerobic central metabolism in Escherichia coli for the absolute aerobic production of succinate. Biotechnol Bioeng.

[B21] Alper H, Miyaoku K, Stephanopoulos G (2005). Construction of lycopene-overproducing E. coli strains by combining systematic and combinatorial gene knockout targets. Nature Biotechnololy.

[B22] Klamt S, Stelling J (2002). Combinatorial complexity of pathway analysis in metabolic networks. Mol Biol Rep.

[B23] Stelling J, Klamt S, Bettenbrock K, Schuster S, Gilles ED (2002). Metabolic network structure determines key aspects of functionality and regulation. Nature.

[B24] Deutscher D, Meilijson I, Kupiec M, Ruppin E (2006). Multiple knockout analysis of genetic robustness in the yeast metabolic network. Nature Genetics.

